# Snorkel: An Epitope Tagging System for Measuring the Surface Expression of Membrane Proteins

**DOI:** 10.1371/journal.pone.0073255

**Published:** 2013-09-02

**Authors:** Michael Brown, Lewis J. Stafford, Dale Onisk, Tony Joaquim, Alhagie Tobb, Larissa Goldman, David Fancy, James Stave, Ross Chambers

**Affiliations:** SDIX, Newark, Delaware, United States of America; NHLBI, NIH, United States of America

## Abstract

Tags are widely used to monitor a protein’s expression level, interactions, protein trafficking, and localization. Membrane proteins are often tagged in their extracellular domains to allow discrimination between protein in the plasma membrane from that in internal pools. Multipass membrane proteins offer special challenges for inserting a tag since the extracellular regions are often composed of small loops and thus inserting an epitope tag risks perturbing the structure, function, or location of the membrane protein. We have developed a novel tagging system called snorkel where a transmembrane domain followed by a tag is appended to the cytoplasmic C-terminus of the membrane protein. In this way the tag is displayed extracellularly, but structurally separate from the membrane protein. We have tested the snorkel tag system on a diverse panel of membrane proteins including GPCRs and ion channels and demonstrated that it reliably allows for monitoring of the surface expression.

## Introduction

Membrane proteins comprise roughly a quarter of the mammalian proteome and perform a wide variety of important functions, but are amongst the most challenging proteins to study [1,2]. Plasma membrane proteins are often difficult to over express in their native state due to their complex synthesis, folding, assembly, and trafficking controls [3]. This is often the case for the therapeutically important G-protein coupled receptors and ion channels [4–6]. The secretory pathway is often the bottleneck in their production and protein over expression can result in most of the protein being trapped inside the cell. To ensure the correct structure of membrane proteins the secretory pathway contains a system of chaperones and quality control mechanisms to check proteins as they pass through [7]. In addition, proteins can contain retention signals that hold them back in the ER or Golgi compartments, or are subjected to trafficking controls that remove them from the plasma membrane [4–6].

The plasma membrane is a critical destination for many membrane proteins where they can interact with the external environment to bind ligands and associate with other proteins. The pool of protein at the plasma membrane contains the fully matured and native structure of the protein that is needed for characterization studies and antibody production for native epitopes [8]. The amount of protein at the plasma membrane can be optimized for these purposes by a variety of manipulations such as choice of transcriptional expression elements, cell lines, and culture media formulation, or by altering the gene by introducing truncations, and other mutations [9]. In general an empirical approach must be taken by systematically testing variables and monitoring surface expression. High surface level expression is especially critical for generating antibodies, either by immunizing with target bearing cells or via DNA immunization [8,10,11]. Similarly for analysis purposes, whether by antibody or in functional studies, it is often advantageous to have high expression of the proteins with a high degree of fidelity in their structure. Antibodies against the extracellular epitopes of the membrane protein are powerful tools for measuring the plasma membrane (surface) expression of a membrane protein [12]. This analysis requires that the surface located protein be distinguished from internal cellular pools, which can be structurally and/or functionally aberrant. The antibodies must be of high specificity to discriminate amongst the thousands of other proteins, and of high sensitivity as many membrane proteins are expressed at low levels. Unfortunately, few antibodies are available that meet these specifications. This is especially problematic for multispan membrane proteins that are much more difficult to raise antibodies against. Instead, tags are often fused to the protein that are detected with antibodies, (HA, FLAG), or other selective reagents, (SNAP, BLAP) [13–16].

A critical step in tagging a membrane protein is to locate a site within the extracellular region where a tag can be inserted without perturbing the structure, function, or sub-cellular localization. This can be particularly challenging with multipass membrane proteins that only have short regions on the surface such as G-protein coupled receptors, ion channels and transporters. The tag insertion site is usually selected empirically and commonly is appended to the N- or C-terminal regions where it is hoped to not perturb the protein [14]. For proteins such as ion channels where the N- and C-termini are located internally, the tag must be inserted into one of the extracellular loops. This is much more challenging and problems can occur ranging from protein instability, misfolding, aberrant post-translational modifications, and functional changes [14,17,18].

While problems with finding suitable tag insertion sites may be empirically solved for functional studies, it still presents a critical problem when the protein is used for antibody production. It is of paramount importance to maintain the natural structure of the antigen in order to generate antibodies that can recognize the native target protein with high affinity. Insertion of a tag into proteins such as GPCRs and ion channels that have only small regions exposed extracellularly makes it highly likely that the resulting antibodies would not necessarily recognize the native protein, create competing epitopes to distract the immune system, and potentially perturb the protein and limit expression. Here we describe the snorkel tag system that creates a separate extracellular region attached to the target protein from which tags can be displayed.

## Methods

### Plasmid construction

pSNKL-Q was designed from a transmembrane domain (residues 530 to 555) from mouse Beta-type platelet-derived growth factor receptor PDGFRB, (Uniprot accession P05622). The sequences naturally flanking the transmembrane domain were altered to conform to the “positive inside” since the topology is reversed [19]. The natural C-terminal region of the PDGFRB (residues 556 to 566) was moved to the N-terminus and at the C-terminus was placed a short linker (GS), a FLAG tag epitope (DYKDDDDK), a short sequence for a SphI restriction site (encoding residues GMQ), the 9 aa HA tag (YPYDVPDYA), and a stop codon. A DNA fragment encoding the snorkel tag with EcoRI and AgeI restriction sites was cloned into the EcoRI and XmaI sites of the expression plasmid pCI (Promega). pSNKL-SNAP was designed by substituting the L2 and tag from pSNKL-Q with the L2 linker GSEYRDEDEKGMQ followed by the SNAP ORF (AFU51890), and HA at the C-terminus. pSNKL-CD24 was designed by substituting the L2 and tag from pSNKL-Q with the L2 linker (GSSGSS) and mouse CD24 ORF (P24807). All membrane protein genes were human protein and built from synthetic oligonucleotides using a codon table with frequently used mammalian codons. With the exception of CD20, Kir2.1, Kv1.3, Kir1.1, TASK3, KCa3.1 all genes had a leader sequence from the human tissue plasminogen activator fused to the N-terminus (MDAMKRGLCCVLLLCGAVFVSPS). A Kozak sequence (GCCGCCACC) was added to the 5’ end of the gene and universal primers were added to both the 5’ (cacttctggtgcttctggc) and 3’ (aagatccgctacttgctcc) ends to allow amplification and dU cloning [20]. Some of the genes contained N- or C-terminal truncations; pre and pro sequence deletions (VIPR1, F2R), or C-termini deletions to remove internalization signals to enhance the surface expression levels of the proteins for subsequent antibody development (VIPR1, ADORA2A, F2R, EP4, CXCR4, LPAR1, GRPR, ADRB2). The regions of the proteins used to construct synthetic genes were; VIPR1 (Uniprot P32241) region 31-402, ADORA2A (Uniprot P29274) region 1-311, F2R (Uniprot P25116) region 42-377, EP4 (Uniprot P35408) region 1-350, CXCR4 (Uniprot P61073) region 1-318 and three phosphorylation sites mutated to alanine (T311A, S312A, T318A), LPAR1 (Uniprot Q92633) region 1-340, GRPR (Uniprot P30550) region 1-343, ADRB2 (Uniprot P07550) region 1-365, CD33 (Uniprot P20138) region 18-364. CD20 (Uniprot P11826), DARC (Uniprot Q16570) Kir2.1 (P63252), Kv1.3 (P22001), Kir1.1 (P48048), TASK3 (Q9NPC2), KCa3.1 (O15554) were all full length. “STOP” versions of genes with no snorkel were constructed by inserting a stop codon in the PCR primer and subcloning back into the snorkel plasmid.

### Cell culture

FreeStyle™ 293-F cells were obtained from Life Technologies. Cells were subcultured as outlined by the manufacturer. Briefly, cells were grown in FreeStyle™ 293 Expression Medium (Life Technologies) in 125 mL shaker flasks. Flasks were seeded at a density of 1x10^5^ viable cells /mL (30 mL final volume). Flasks were incubated in a humidified incubator at 37^o^C, 8% CO_2_ on an orbital shaker platform rotating at 130 rpm. Cell density and viability was monitored and cells were sub-cultured when the density reached 1x10^6^ viable cells/mL.

### Transfections

Twenty-four hours before the transfection, the 293-F cells were sub-cultured at a density of about 6x10^5^ cells/mL. The day of transfection, the viability of the cells was determined to be >90% and the cells were diluted to a density of 1x10^6^ cells/mL and 30 mL was placed in each 125 mL shaker flask. The plasmid DNA was diluted as recommended for the FreeStyle™ 293-F cells. Briefly, 37.5 µg of DNA was added to OptiPro™ SFM to a final volume of 0.6 mL and mixed. In a second tube 37.5 µL of Life Technlologies’ FreeStyle™ MAX reagent was added to a total OptiPro™ SFM final volume of 0.6 mL and mixed by inversion. The contents of the two tubes were incubated for 5 minutes. The two tubes were then mixed and incubated for 30 minutes at room temperature. The mixture was added slowly with swirling to the flask containing the cells. The flask was incubated at 37^o^C, 8% CO_2_ on an orbital shaking platform rotating at 130 rpm.

### Western blot

One million transfected HEK293 cells were centrifuged at 3000 rpm for 1minute in a microfuge, the cell pellet was resuspended with 200µl of extraction buffer, (1% Triton X-114, 25mM Tris pH 8.0, 1mM EDTA and 0.2mg/ml protease inhibitors (Boehringer), centrifuged at 3000 rpm for 1minute and the supernatant collected. The protein extracts were electrophoretically separated under denaturing conditions on SDS-PAGE, 4-20% Tris-HCl pre-cast gels in a Criterion Cell apparatus, (Bio-Rad), alongside MagicMark XP protein markers (Life Technologies) in running buffer as recommended by the manufacturer (Bio-Rad). Fractionated proteins were electro-blotted onto nitrocellulose membranes (0.45 µm pore size, Protran, Whatman) in a semi-dry electrophoretic transfer cell unit (Trans-Blot^®^ SD, Bio-Rad) and then blocked with TBST (Sigma) supplemented with 2% Difco skim milk (Becton Dickinson). HA tagged proteins were detected with a rabbit anti-HA antibody (Bethyl) and a goat anti-rabbit antibody conjugated to HRP (Jackson Immunoresearch). Blots were then developed with chemiluminescent substrate (SuperSignal^®^ West Femto, Thermo Scientific) and the signal was captured with a multi-purpose Image Station 440CF system (ver. 3.6, Eastman Kodak).

### Flow Cytometry

Flow cytometry was performed on a Guava EasyCyte Plus (Millipore). Briefly, 2-5 x 10^4^ transfected cells were placed in each well of a 96 well V bottom plate and stained with saturating amounts of fluorescently labeled monoclonal antibodies (FITC or phycoerythrin (PE)) Anti HA (Miltenyi Biotec); PE CD20, and PE Anti DARC (R&D Systems); PE Anti CXCR4 (Biolegend); PE anti-CD24 (BD Bioscience), or PE labeled isotype controls (Santa Cruz Biotechnology). For SNAP tag staining we used SNAP surface Alexa fluor 488 (NEB). All staining was in a final volume of 50 µl of 10% normal goat serum (heat inactivated, 30 minutes at 56^o^C) in PBS with 0.025% sodium azide and was performed at 2-8^o^C. After 30 minutes with gentle shaking cells were washed three times with cold 1% bovine serum albumin (BSA) in PBS with 0.025% sodium azide and analyzed. Flow cytometer calibration was performed using Rainbow Calibrator Particles RCP 30-5A (Spherotech).

For surface staining, only viable cells as judged by their light scatter characteristics (forward angle and side scatter) were gated to be included in the analysis. Total staining (surface plus internal) using was performed using the Fix and Perm Cell Permeabilization Kit (Life Technologies) as follows: Duplicate wells were stained as described previously. After staining and washing one well of the replicate was fixed using 50 µl of the kit medium A for 30 minutes at room temperature. After washing cells were resuspended in 50 µl of kit medium B to permeabilize the cells and antibody again added. After staining for 30 minutes 2-8^o^C with gentle shaking cells were washed with cold 1% BSA in PBS, 0.025% sodium azide and 0.1% saponin to facilitate washing and analyzed, along with the replicate that received only the surface staining. FRET was performed with the 743AB30 anti-CD20 mouse monoclonal antibody (SDIX), rabbit anti-HA antibody labeled with Alexa488 (Bethyl), and goat anti-mouse antibody labeled with Alexa647 (Jackson Immunoresearch). All dual staining experiments were fully compensated to eliminate contribution of spectral bleedover.

### Ligand Binding by CXCR4 Snorkel constructs

SDF-1 (PeproTech, Rocky Hill, NJ) was labeled with NHS-LC-Biotin (Thermo) at pH 8.5 for 30 minutes at room temperature using a 5: 1 molar excess of NHS-LC-Biotin. For binding studies 5E4 HEK293 cells transfected with CXCR4-snorkel, or control were incubated for 1 hour at 4 degrees in with biotinyl SDF-1 at a final concentration of 10 nM. Cells were then washed three times at 4 degrees and incubated an additional 30 minutes with 15nM PE labeled streptavidin (Jackson Immunoresearch, West Grove, PA). After additional washing cells were analyzed by flow cytometry. For antibody blocking experiments monoclonal antibodies to CXCR4 or CD20 as a control were added to cells at 10 nM at 4 degrees for 30 minutes prior to the addition of biotinyl SDF with no washing between these two steps.

## Results

**Figure 1:**
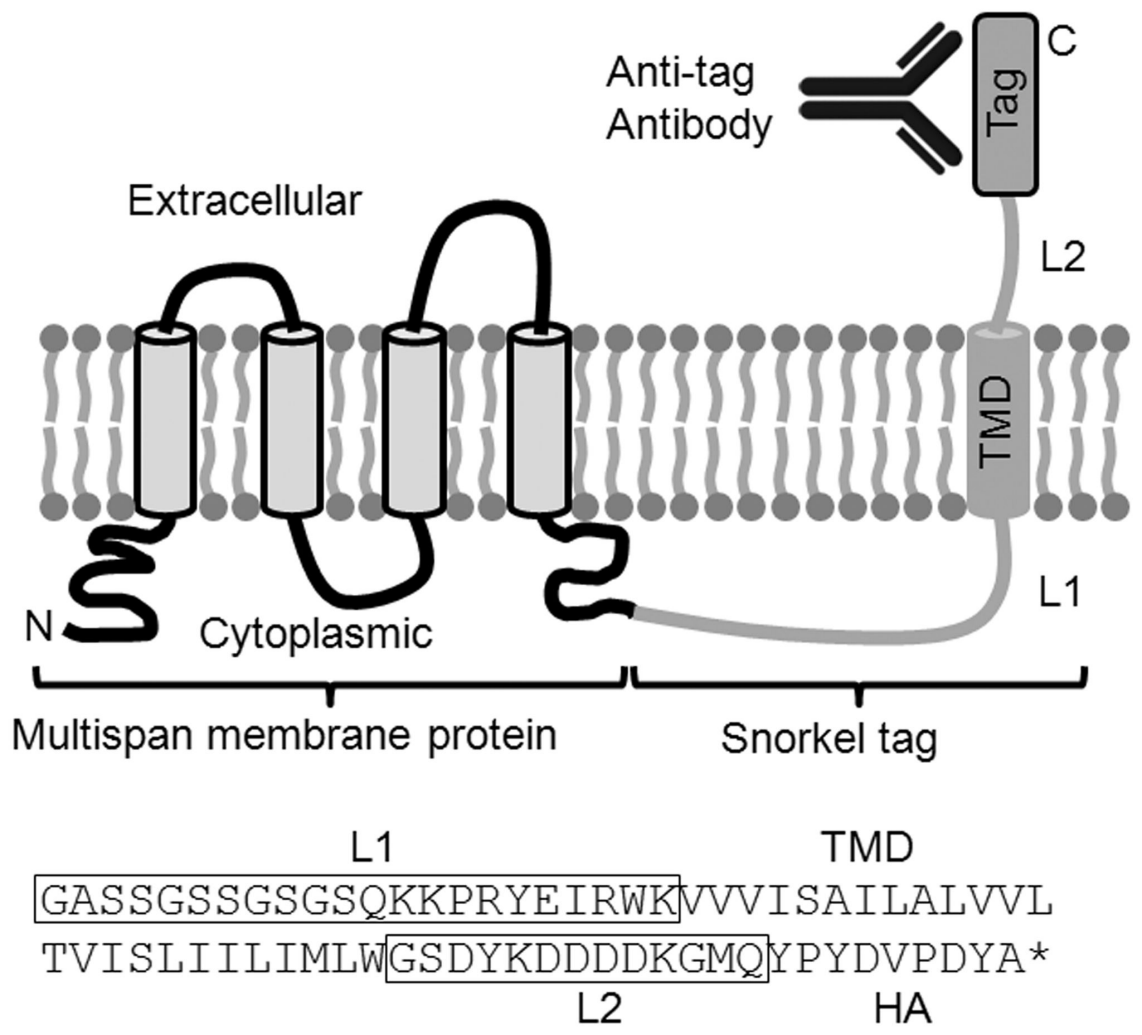
Design of the snorkel tag. A model four span multipass membrane protein is shown in black with its C-terminus linked to the snorkel tag. L1 = linker 1, TMD = transmembrane domain, L2 = linker 2.

Our goal was to create a routine way of constructing tagged membrane proteins that would allow the measurement of their surface expression level, while minimizing perturbation to their structure, function, or location. Our “snorkel” design was an epitope tag and a transmembrane domain that are fused to a membrane protein (Figure 1). The pSNKL-Q snorkel plasmid was designed to be fused to cytoplasmically located C-termini as this is the most common topology in membrane proteins (>75%), including GPCRs and ion channels [2]. In this way, the epitope tag is displayed extracellularly, but is not embedded in the extracellular regions of the target protein where it could influence structure and/or function. The SNKL-Q ORF is composed of a 22 residue linker (L1), a 25 residue transmembrane domain (TMD), a 13 residue linker (L2), and a 9 residue HA epitope tag.

**Figure 2:**
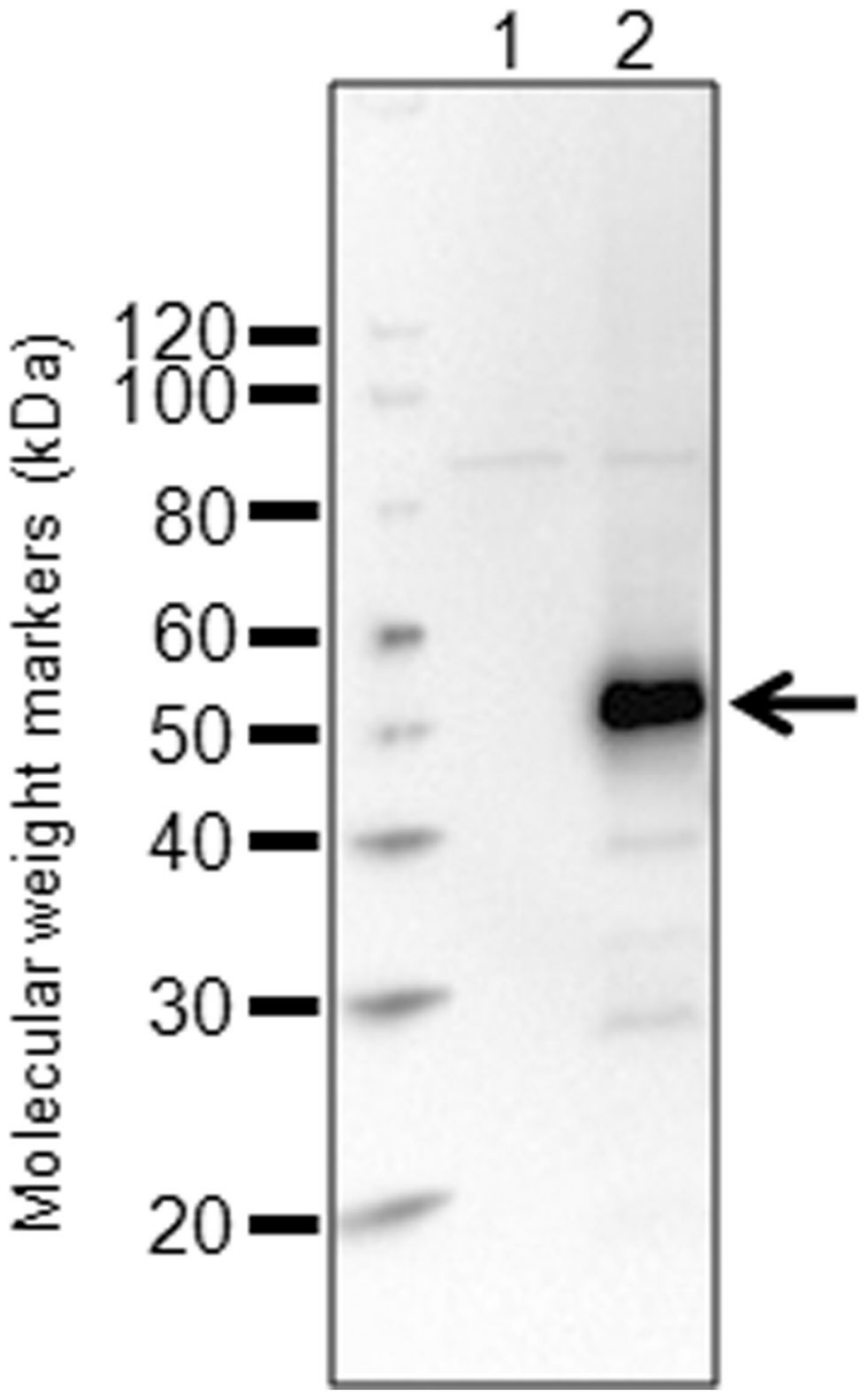
Western blot analysis of the snorkel tag construct. Plasmid constructs were transiently transfected into HEK293 cells, grown for 22h before analysis in western blot. A. Western blot with an anti-HA antibody. Lane 1; CD20 with a stop codon before the snorkel tag, lane 2; CD20 fused to the snorkel tag containing the HA epitope tag.

The SNKL-Q design was evaluated as a tagging method with the four-span membrane protein CD20 that contains two extracellular loops, 6 and 47 residues, similar to the cartoon shown in Figure 1. Epitope tagging the extracellular region would normally require insertion of the epitope tag into the very small loops that would significantly disrupt the structure and possibly function. The human CD20 gene was cloned into the pSNKL-Q plasmid to either fuse the C-terminus in frame to the SNKL-Q ORF, or with a stop codon inserted immediately after the CD20 ORF (STOP version). The plasmids were transiently transfected into HEK293 cells and allowed to express the tagged proteins. Whole cell extracts from the cells were analyzed by western blotting using an anti-HA antibody that detects the HA epitope present in SNKL-Q. A single predominant band of ~50 kDa was detected in the SNKL-Q construct with minimal other bands, whereas as expected the CD20 STOP version showed no significant staining (Figure 2).

**Figure 3:**
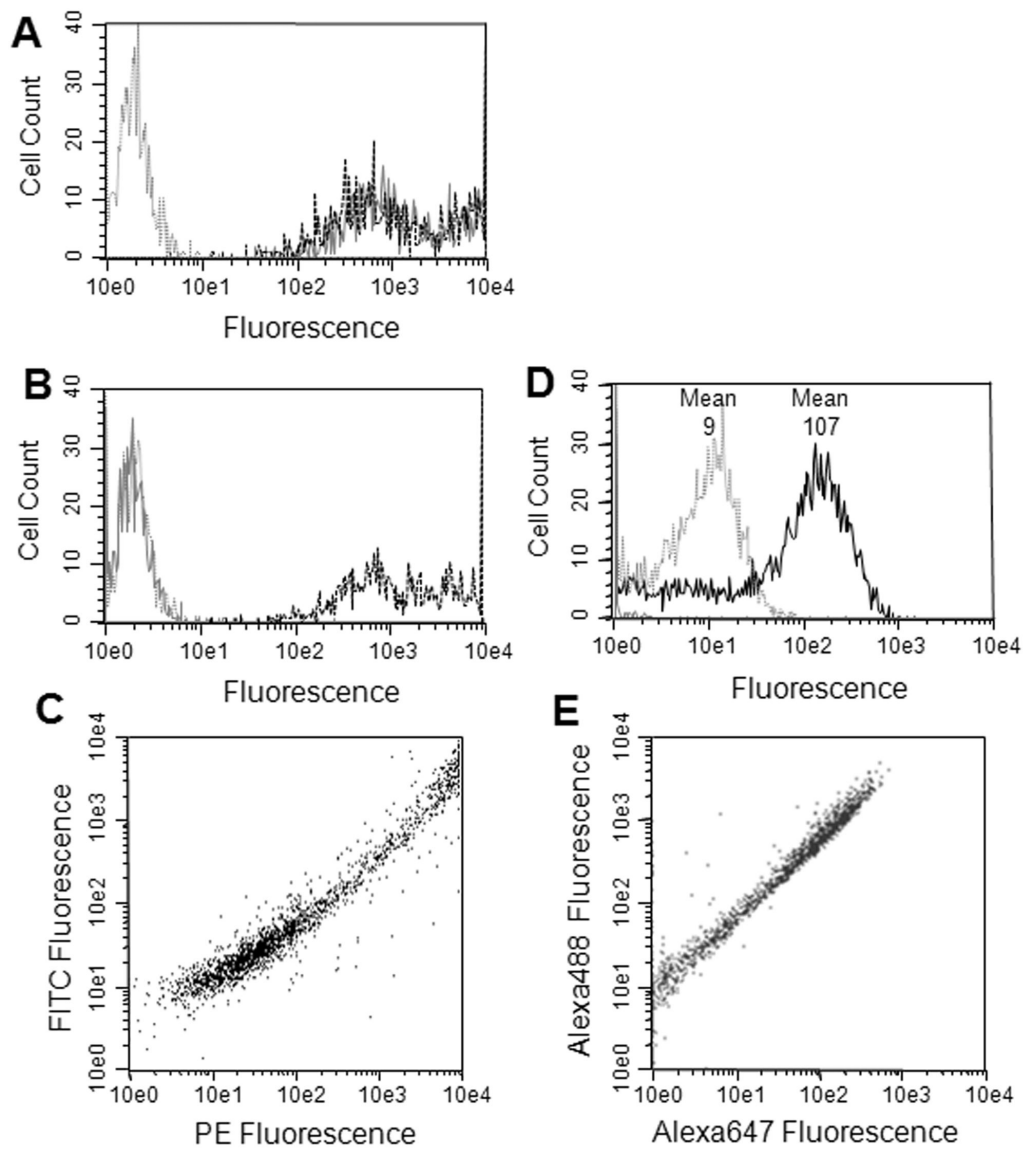
Correlating membrane protein and snorkel tag expression. Plasmid constructs were transiently transfected into HEK293 cells, grown for 22h before analysis in flow cytometry. A. Flow cytometry with CD20-snorkel cells. Transfected cells stained with anti-HA antibody (grey solid line) or with anti-CD20 (black dashed line); Grey dashed line (control); untransfected cells stained with an anti-HA antibody. B. same as A except with a CD20-stop construct. C CD20-snorkel dual stained with anti-HA (FITC labeled) and anti-CD20 (PE labeled). D. CD20-snorkel stained with CD20 antibody and the Alexa647 goat anti-mouse antibody (grey line), FRET measurement with the CD20-snorkel construct stained with anti-HA (Alexa488 labeled) and anti-CD20 and goat anti-mouse (Alexa647 labeled, black line). E same as D but plotted with both Alexa488 and 647 fluorescence for FRET pair.

Flow cytometry was performed on the transfected cells stained with either anti-HA or anti-CD20 antibodies. Significant staining was observed on the CD20 SNKL-Q transfected cells with both antibodies indicating that the snorkel construct was displayed on the surface as expected. Transient transfection typically results in cells with a range of expression levels and both the anti-CD20 and anti-HA antibodies showed very similar patterns of staining across the range of expression levels (Figure 3A). In contrast, the CD20 STOP version stained with anti-CD20, but not with anti-HA antibodies (Figure 3B). The staining with the CD20 antibody was very similar between the SNKL-Q and STOP versions of CD20 transfected cells indicating that the snorkel did not significantly influence the CD20 expression level. Dual staining the cells transfected with the CD20 pSNKL-Q construct with both anti-HA (FITC labeled) and anti-CD20 (PE labeled) showed the staining of the two labels were highly correlated on individual cells (Figure 3C).

FRET was used to demonstrate that both the CD20 and the snorkel tag remained closely connected extracellularly. A rabbit anti-HA antibody labeled with Alexa488 was used to label the snorkel tag and a CD20 mouse monoclonal antibody with a goat anti-mouse secondary antibody labeled with Alexa647 was used to label the CD20 extracellular epitope. Cells stained with Alexa488 Anti-HA showed very little background in the red fluorescence (FRET) channel, and slightly higher red fluorescence, (mean = 9), was seen with cells stained with the CD20 antibody and the Alexa647 goat anti-mouse antibody (Figure 3D). However, when both sets of antibodies were used to stain cells a >10-fold increase in red fluorescence, (mean = 107), was observed indicating a FRET between the closely associated CD20 and snorkel HA epitopes (Figure 3D). Moreover, on individual cells the FRET red fluorescence was tightly correlated with the donor green fluorescence (Figure 3E).

**Figure 4:**
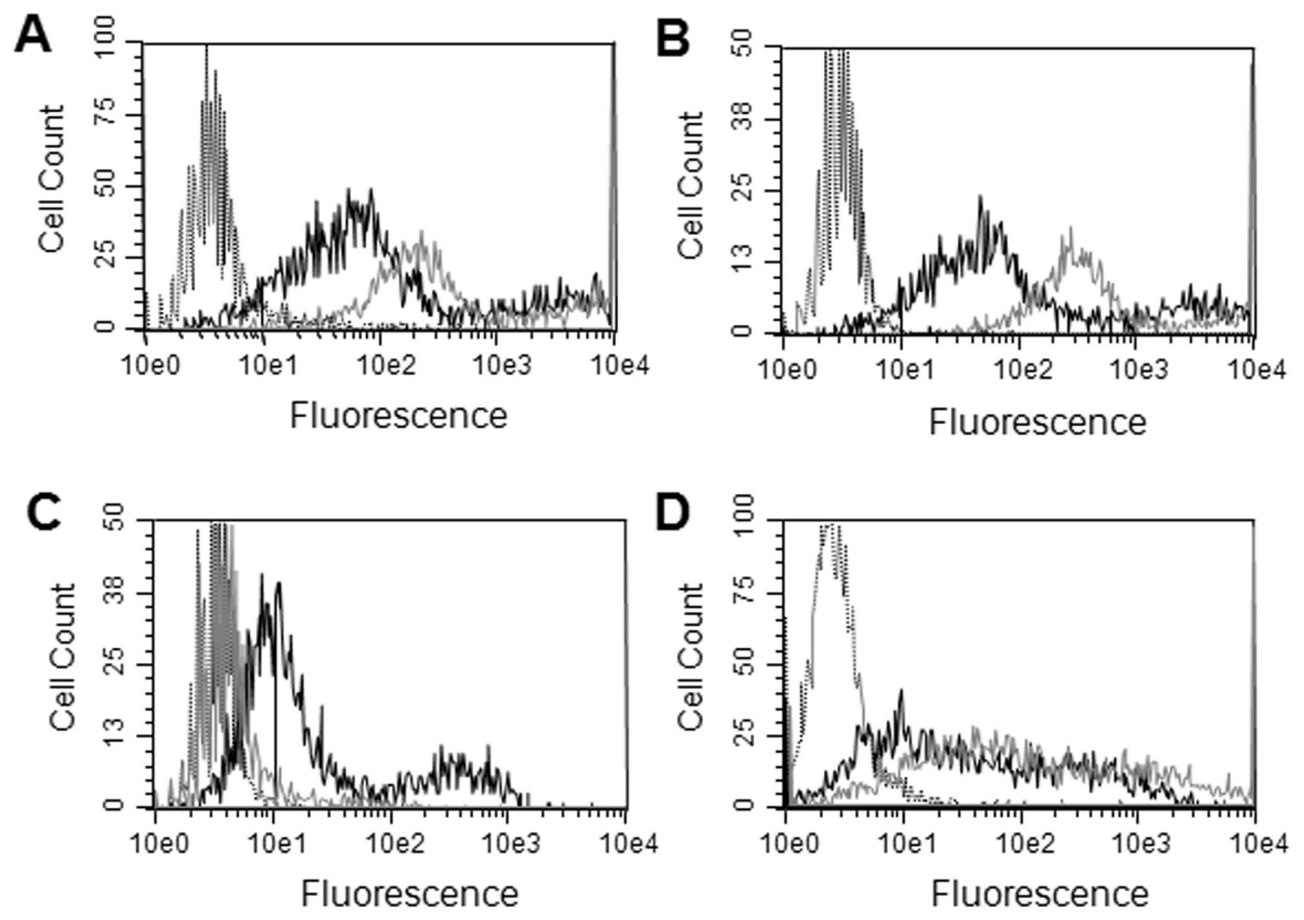
SNAP and CD24 snorkel constructs. CD20 plasmid constructs were transiently transfected into HEK293 cells, grown for 22h before analysis in flow cytometry. A. Staining with a CD20 antibody, grey solid line; pSNKL-Q construct, black solid line; pSNKL-SNAP construct, dashed black line; non-transfected cells. B. same as A but stained with anti-HA antibody. C. same as A but with stained with the SNAP labeling reagent. D. CD20 in pSNKL-CD24. Black solid line; stained with CD20 antibody, grey solid line; stained with CD24 antibody, black dashed line; stained with HA antibody.

Snorkel plasmids with other epitope tags, (myc and FLAG), as well as different L2 linkers were constructed and evaluated and worked similarly as pSNKL-Q (unpublished observations). To investigate the ability of the snorkel to display a much larger tag a snorkel was constructed that included the 182 residue SNAP tag (pSNKL-SNAP) in addition to the HA tag. Plasmids encoding CD20 in both pSNKL-Q and pSNKL-SNAP were transfected into HEK293 cells and the expression of the tags monitored by flow cytometry. Staining cells with either the CD20 or HA antibody both showed that the constructs displayed HA, albeit the pSNKL-SNAP showed lower expression than pSNKL-Q (Figure 4A & 4B). Staining the pSNKL-SNAP transfected cells with the non-permeant fluorescent substrate showed that the SNAP tag was displayed in a functional form (Figure 4C). In an additional example mouse CD24 was repurposed as an epitope tag, (validated commercial CD24 antibodies are available), and a snorkel plasmid constructed by encoding the mouse CD24 gene as the epitope tag. The extracellular region of pSNKL-CD24 was 33 residues long, (L2 linker and mouse CD24), and includes multiple glycosylation sites. CD20 was cloned into pSNKL-CD24, transfected into HEK293 cells and stained with CD24 (mouse), CD20, and HA antibodies. Both the CD20 and CD24 antibodies stained the transfected cells in a similar manner, and as expected no staining was seen with the HA antibody (Figure 4D).

A panel of 14 genes encoding human multipass membrane proteins were cloned into the SNKL-Q plasmid and used to determine the ability of the snorkel tag to work with a range of different proteins. The genes were selected to be relatively diverse and with a range of expression levels. The genes included a diverse panel of GPCRs; VIPR1, ADORA2A, F2R, EP4, CXCR4, LPAR1, GRPR, ADRB2 and DARC, and a panel of ion channels; TASK3, Kir2.1, Kv1.3, KCa3.1, and Kir1.1. All of the proteins had topologies that had been annotated to have their C-termini located in the cytoplasm. The resulting SNKL-Q plasmids were transiently transfected into HEK293 cells and were analyzed by flow cytometry with an anti-HA antibody, and where available anti-target antibodies. Since some of the proteins are known to become trapped intracellularly, the cells were stained with and without permeabilization to detect internal pools of the proteins.

**Figure 5:**
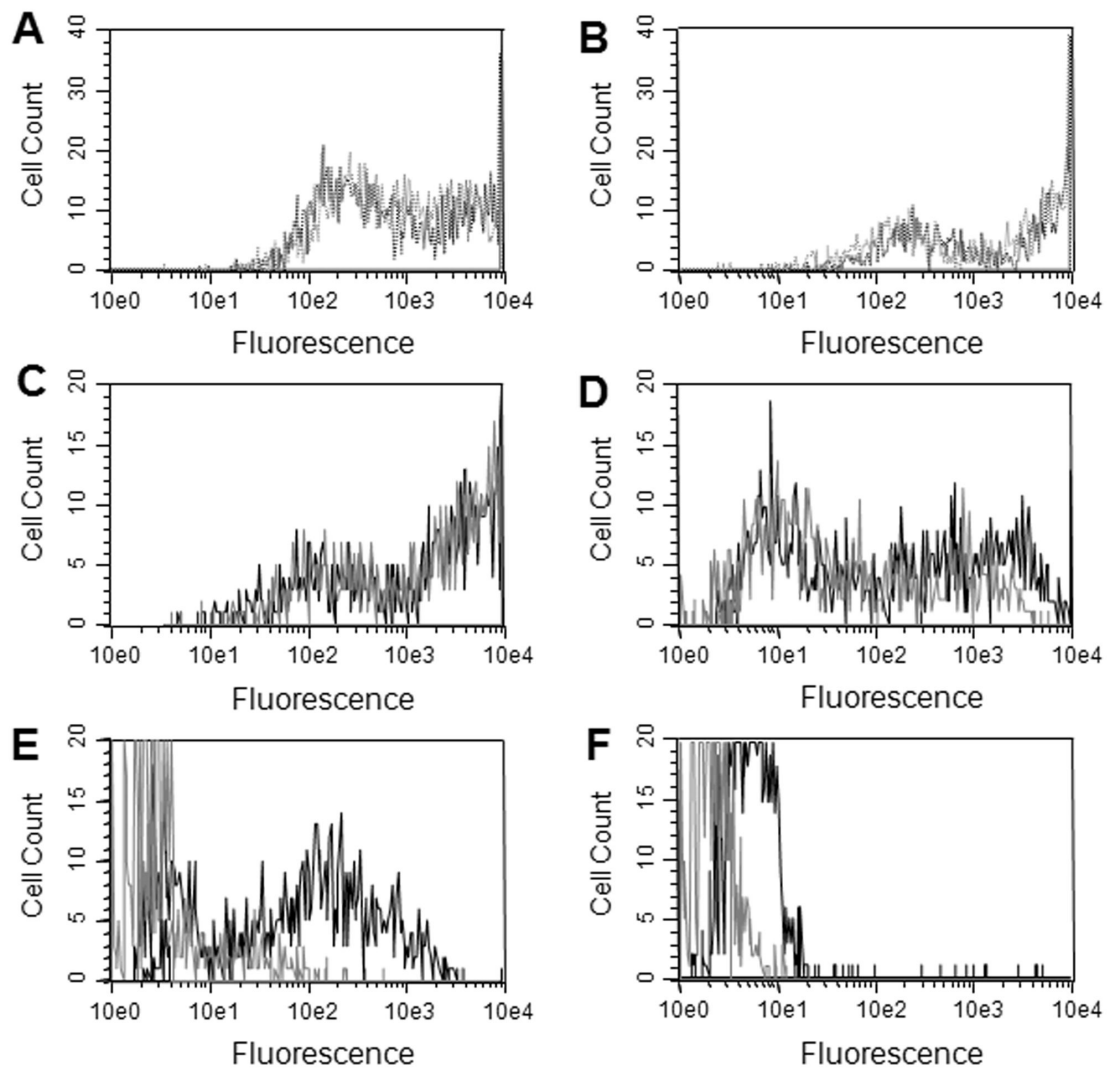
Evaluation of the snorkel tag with a panel of membrane proteins. Plasmid constructs based on pSNKL-Q with different membrane protein genes were transiently transfected into HEK293 cells, grown for 22h before analysis in flow cytometry. A. CXCR4, black dashed line; anti-CXCR4 antibody, grey dashed line; anti-HA antibody. B. same as A but with DARC and anti-DARC antibody. C through F were all stained with anti-HA antibodies either surface staining only (grey solid line), or with permeabilized cells (black solid line). C. CD20, D. Kir2.1, E. Kv1.3, F. Kir1.1.

Relatively high levels of staining were observed for all the cells transfected with GPCR genes with only a slight increase in staining with permeabilization, indicating that most of the protein was located on the plasma membrane (Figures 5 & S1). Commercial antibodies suitable for flow cytometry were available for CXCR4 and DARC and were used to compare with the snorkel tag staining. Similar staining profiles were seen between the CXCR4 and DARC antibodies and the anti-HA antibodies (Figure 5A & 5B). The snorkel tagged CXCR4 was also able to bind the natural CXCR4 ligand SDF-1 (Figure S2). Kir2.1, CD20, VIPR1, ADORA2A, F2R, EP4, LPAR1, GRPR, and ADRB2 all showed a relatively high surface staining with the snorkel tag with little trapped in intracellular pools (Figure 5C, 5D, & S1). Kv1.3, TASK3, and KCa3.1 showed little surface expression with the majority present in intracellular pools (Figure 5E & S1). Kir1.1 showed little to no expression either intracellularly or on the surface (Figure 5F). By way of reference, calibrator beads with 29,000, 127,000, and 293,000 equivalents of PE equivalents gave fluorescent peaks with mean channels at 80, 318, and 739 using the same instrument setup.

## Discussion

Measuring the surface expression of multispan membrane proteins can be challenging either because of the unavailability of antibodies, inserting a tag in the small extracellular loops perturbs the structure, function, or location, or finding a site that is accessible for the tag labeling reagents. We have developed the snorkel tag that creates a structurally separate extracellular region from which to display tags. The snorkel is composed of four parts; linker L1, transmembrane domain, linker L2, and the tag. The transmembrane domain was derived from PDGFRB, a type I membrane protein. In the snorkel construct the topology of the transmembrane domain is reversed from the native topology in PDGFRB. We therefore altered the membrane flanking residues in L1 to conform to the “positive inside” rule to ensure extracellular display of the tag [19]. We investigated the use of a transmembrane domain from the TRF1 protein where the transmembrane domain is naturally in the desired topology, (i.e. a type II membrane protein), but the snorkel constructs gave much poorer expression levels compared with the PDGFRB-based construct (unpublished observations).

The topology of the tag in the CD20 snorkel constructs was investigated with anti-HA antibodies and confirmed that it was indeed located extracellularly and was accessible to antibodies. Dual staining with CD20 and HA antibodies labeled with different fluorophores showed a close correlation of fluorescence between the HA tag and the CD20 protein on individual cells (Figure 3C). This relationship extended over three orders of magnitude in expression indicating the snorkel was not impeding synthesis and maturation even at high levels. This does not exclude the possibility of whether the snorkel fusions generate a mixture of topological states with different molecules displaying either the CD20 epitopes or the tag epitope but not both together. However, FRET experiments between the CD20 and the tag indicated that the extracellular regions of CD20 and the snorkel are almost exclusively displayed together in the same molecule (Figure 3E). Similar results were obtained with CXCR4 and ADORA2a (data not shown). Western blot analysis showed little evidence of proteolysis of the snorkel fusion constructs (Figure 2). While exact quantitation in flow cytometry can be problematic [21], it should be noted that a significant number of cells in these heterogeneous populations induced by transient transfection showed staining in excess of the level observed with calibrator beads with 293,000 equivalents of PE, suggesting very high expression levels at the cell surface. Titering the antibody conjugates to saturation and use of equimolar conjugates of antibody and PE would be required for exact quantitation [22]. Nonetheless expression levels observed were assumed to be quite high.

The snorkel extracellular region was originally designed with the HA epitope tag (pSNKL-Q). We have made and tested a variety of different snorkel constructs with different L2 linkers and different tags (Figure 4). Alternate small epitope tags and alterations to the L2 linker sequence with extracellular snorkel regions of <40 residues, all performed similarly to pSNKL-Q (unpublished observations). FLAG tag is also present in pSNKL-Q within the L2 linker and 2 residues from the transmembrane domain. However, FLAG tag in pSNKL-Q stained poorly, but worked significantly better in a different design where it was positioned 13 rather than 2 residues from the transmembrane domain (unpublished observations).

The largest tag evaluated was the SNAP tag which together with the L2 linker and a HA tag created an extracellular snorkel region of 204 residues. The pSNKL-SNAP construct expressed at lower levels compared with the smaller pSNKL-Q construct (Figure 4). However, the SNAP tag that was expressed was functional as evidenced by its ability to covalently label with the SNAP substrate. We explored the use of an alternative epitope tag with the use of mouse CD24 and showed it could function as an epitope tag (Figure 4D). One important application of overexpressing membrane proteins is the generation of antigen for antibody development. We have demonstrated with DNA immunization that the HA tag within pSNKL-Q is as expected highly immunogenic and can dominate the immune response especially with weakly immunogenic proteins such as highly conserved proteins (unpublished observations). Mouse CD24 was selected as an epitope tag as it is very small, (27 residues), there are validated antibody reagents commercially available, and mice should suppress the anti-CD24 immune response due to immune tolerance.

Several lines of evidence indicate that the snorkel tag does not significantly alter the structure of the attached membrane protein. Major structural changes would likely result in misfolding and trapping of the protein in the endoplasmic reticulum by the ERAD system. We can largely rule this out at least in the cases of CD20 and the GPCRs where we did not see significant amounts of the proteins trapped inside the cell. It is less clear for TASK3, KCa3.1 and Kv1.3 since most of the protein appeared to be trapped inside the cell, or in the case of Kir1.1, not expressed at all. However, these ion channels have been described previously as being difficult to express on the surface of cells due to problems with folding, assembly and/or trafficking [23–26]. A second line of evidence comes from the staining with antibodies to CD20, CXCR4, and DARC which did not show differential staining of their cognate antigens with or without the snorkel. Moreover, we have generated panels of >50 antibodies against CD20 and CXCR4 that map to a wide number of epitopes and they did not show differential staining with and without the snorkel (unpublished observations). Adding extracellular tags to membrane proteins via an additional transmembrane domain has also been reported with the ion channels TRPV5 [27] and CFTR [28]. In these cases the tags were fused to the N-terminus and yielded functional proteins that behaved normally.

One of the potential limitations with the snorkel design is steric constraints imposed by the L1 linker that links the C-terminus of the protein to the snorkel transmembrane domain resulting in misfolding. All of the snorkel constructs described here used the same L1 linker which was 22 residues long. It is composed of an 11 residues glycine/serine rich sequence and the 11 aa region from PDGFRB that is naturally adjacent to the membrane. Assuming the L1 linker is linear it could maximally stretch a relatively large distance of ~80 Angstroms. The presence of the snorkel in the CXCR4 construct did not interfere with a panel of antibodies binding to CXCR4 epitopes, nor to the binding of the ligand SDF-1. Within the panel of membrane proteins tested here the size of their cytoplasmic regions ranged from 9 to 249 residues. We did not see a correlation between the size of the C-terminal cytoplasmic domain and snorkel expression level nor intracellular trapping. The largest C-termini tested here (Kir2.1, 249 residues) showed high levels of surface snorkel staining (Figure 5D). Increasing the length of the glycine/serine linker could address cases where the C-termini are located at much larger distances from the membrane. We have tested L2 linkers with glycine/serine linkers of up to 25 residues and seen similar performance (unpublished observations). One potential downside of fusing the snorkel tag to the C-terminus is interference with proteins that bind to this region, such as PDZ binding motifs. While we have not investigated this possibility, in principle the snorkel tag could be adapted to the N-terminus, provided the N-terminus is located in the cytoplasm.

The snorkel tag was developed to allow the routine measurement of surface expression of membrane proteins without extensive trial and error in finding a suitable insertion site. There are several special applications where the snorkel tag may prove especially useful. In cases where two different membrane proteins are being directly compared, the snorkel would provide a more reliable comparison without complications from the influence of local context when a tag is directly linked to the extracellular regions of the protein [18]. This would also simplify the development of FRET assays for measuring ligand binding or receptor multimerization [16].

## Supporting Information

Figure S1 **Evaluation of the snorkel tag with a panel of membrane proteins.** Plasmid constructs based on pSNKL-Q with different membrane protein genes were transiently transfected into HEK293 cells, grown for 22h before analysis in flow cytometry. A through I were all stained with anti-HA antibodies either surface staining only (grey solid line), or with permeabilized cells (black solid line). A. VIPR1, B. ADORA2A, C. F2R, D. EP4, E. LPAR1, F. GRPR, G. ADRB2, H. TASK3, I. KCa3.1.
(TIF)

Figure S2 **Binding of the CXCR4 ligand SDF-1 to a CXCR4-snorkel fusion construct.** The CXCR4-snorkel construct (pSNKL-Q) was transiently transfected into HEK293 cells, grown for 22h before analysis in flow cytometry. Cells were stained with biotinylated SDF-1 followed by streptavidin-PE (grey solid line). As a control, SDF-1 binding was blocked by preincubating the cells with an antagonist CXCR4 antibody (black solid line), or with a control antibody to the unrelated protein CD20 (dashed black line).
(TIF)
