# Supplementary figures and images for: Snorkel: An Epitope Tagging System for Measuring the Surface Expression of Membrane Proteins
Source: PLoS One. 2013 Sep 2;8(9):e73255. doi: 10.1371/journal.pone.0073255 (PMC3759426; doi:10.1371/journal.pone.0073255)

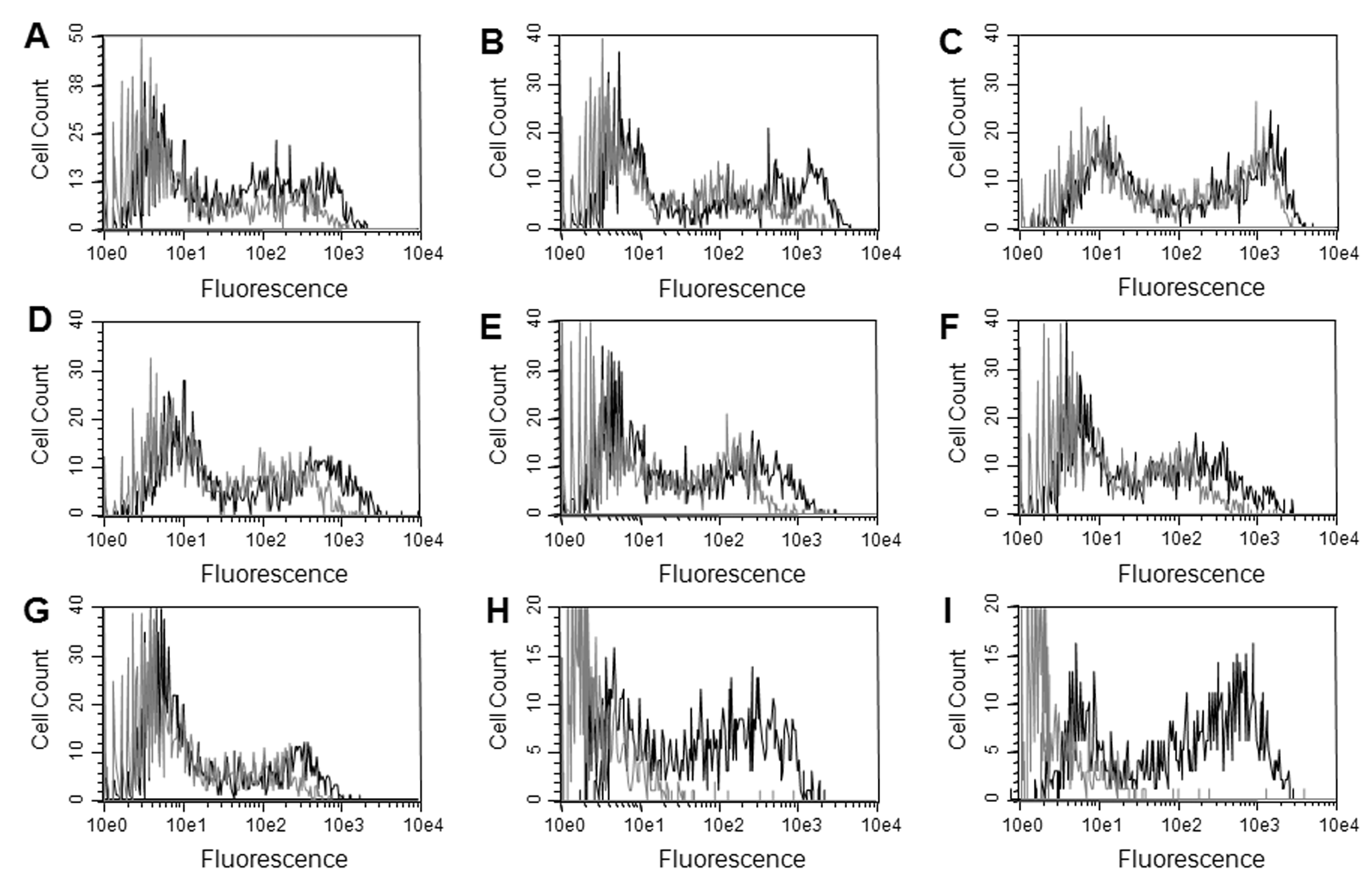

Supplement: Figure S1 — Evaluation of the snorkel tag with a panel of membrane proteins. Plasmid constructs based on pSNKL-Q with different membrane protein genes were transiently transfected into HEK293 cells, grown for 22h before analysis in flow cytometry. A through I were all stained with anti-HA antibodies either surface staining only (grey solid line), or with permeabilized cells (black solid line). A. VIPR1, B. ADORA2A, C. F2R, D. EP4, E. LPAR1, F. GRPR, G. ADRB2, H. TASK3, I. KCa3.1. (TIF) [file pone.0073255.s001.tif]

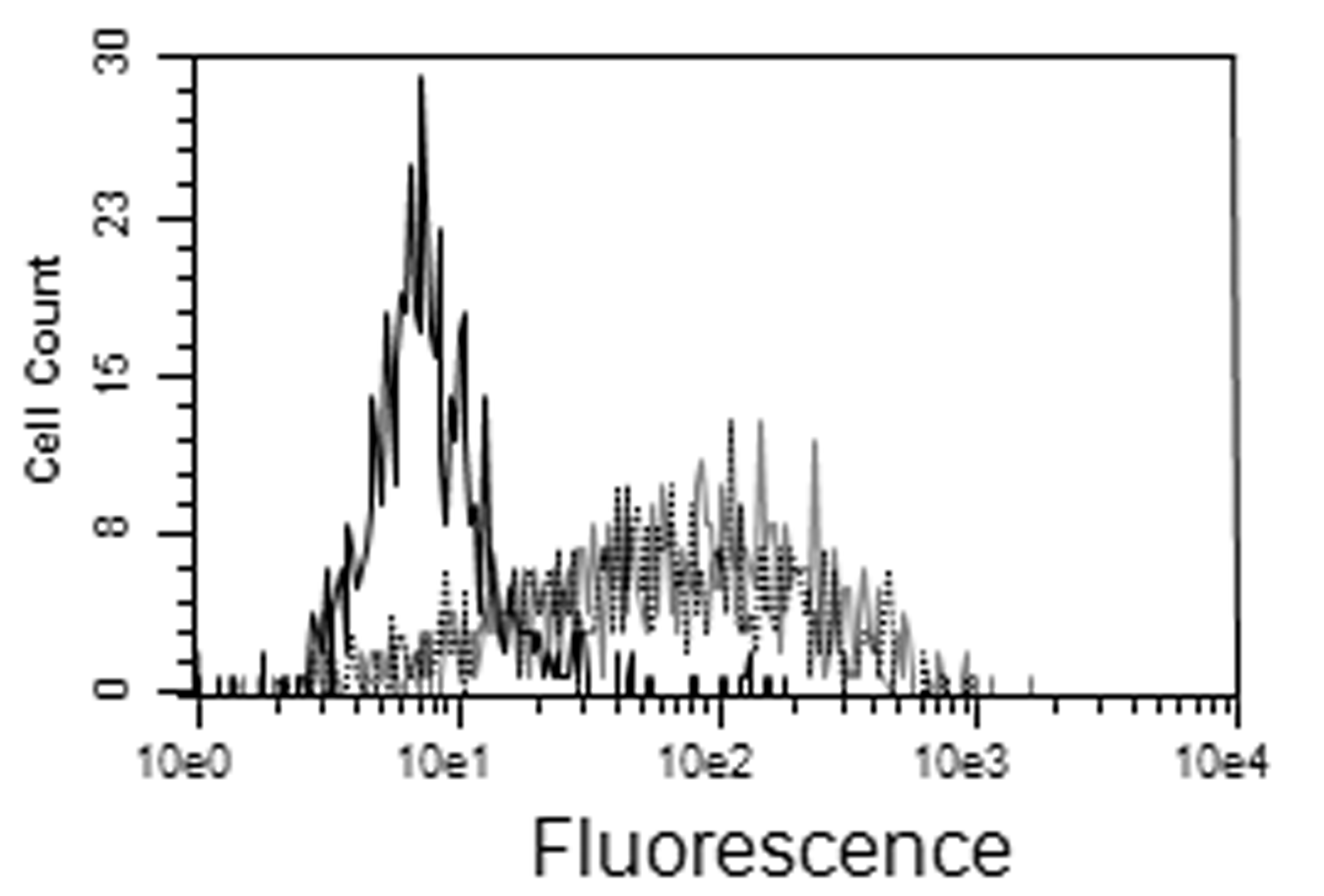

Supplement: Figure S2 — Binding of the CXCR4 ligand SDF-1 to a CXCR4-snorkel fusion construct. The CXCR4-snorkel construct (pSNKL-Q) was transiently transfected into HEK293 cells, grown for 22h before analysis in flow cytometry. Cells were stained with biotinylated SDF-1 followed by streptavidin-PE (grey solid line). As a control, SDF-1 binding was blocked by preincubating the cells with an antagonist CXCR4 antibody (black solid line), or with a control antibody to the unrelated protein CD20 (dashed black line). (TIF) [file pone.0073255.s002.tif]
